# Synthesis and characterization of MoS_2_-carbon based materials for enhanced energy storage applications

**DOI:** 10.1038/s41598-024-77545-w

**Published:** 2024-10-30

**Authors:** Mariusz Szkoda, Anna Ilnicka, Konrad Trzciński, Zuzanna Zarach, Daria Roda, Andrzej P. Nowak

**Affiliations:** 1 Faculty of Chemistry, Department of Chemistry and Technology of Functional Materials, Gdańsk University of Technology, Narutowicza 11/12, Gdańsk, 80-233 Poland; 2 Advanced Materials Center, Gdańsk University of Technology, Narutowicza 11/12, Gdańsk, 80-233 Poland; 3 Faculty of Chemistry, Nicolaus Copernicus University in Torun, Gagarina 7, Toruń, 87-100 Poland

**Keywords:** Energy storage, MoS_2_-based materials, Supercapacitor, Li-ion battery, Na-ion battery, K-ion battery, Batteries, Supercapacitors

## Abstract

The article delves into the synthesis and characterization of MoS_2_-carbon-based materials, holding promise for applications in supercapacitors and ion batteries. The synthesis process entails the preparation of MoS_2_ and its carbon hybrids through exfoliation, hydrothermal treatment, and subsequent pyrolysis. Various analytical techniques were employed to comprehensively examine the structural, compositional, and morphological properties of the resulting materials. The article explores the electrochemical performance of these electrode materials in supercapacitors and ion batteries (LiB, SiB, KiB). Electrochemical measurements were conducted in aqueous electrolyte for supercapacitors and various aprotic electrolytes for ion batteries. Results highlight the impact of the synthesis process on electrochemical performance, emphasizing factors such as capacitance, rate capability, and charge/discharge cycle performance. Hydrothermally treated MoS_2_-carbon exhibited a specific capacitance of approximately 150 F g^-1^ in supercapacitors, attributed to its high surface area and efficient charge storage mechanisms. Additionally, for Li-ion battery materials without hydrothermal treatment showed impressive capacity retention of around 88% after 500 charge-discharge cycles, starting with an initial specific capacity of about 920 mAh/g. Long-term stability was demonstrated in both supercapacitors and lithium-ion batteries, with minimal capacitance degradation even after extensive charge-discharge cycles. This research underscores the potential of MoS_2_-based materials as effective energy storage solutions.

**Supplementary Information:**

The online version contains supplementary material available at 10.1038/s41598-024-77545-w.

## Introduction

Transition metal dichalcogenides (TMDs) have attracted significant attention in recent years due to their unique electronic, optical, and electrochemical properties, making them promising candidates for various applications, including energy storage devices^1,2^. Among the TMDs, molybdenum disulfide (MoS_2_) has emerged as a particularly attractive material owing to its layered structure and remarkable performance in energy storage systems^3,4^. MoS_2_, a typical two-dimensional (2D) layered inorganic material with significant interlayer spacing, has garnered substantial interest in the field of energy conversion and storage applications^5–8^. In recent years, MoS_2_ has been the subject of extensive research, mainly due to its exceptional performance in supercapacitors^9^, photocatalytic applications^10,11^, electrocatalysis^12,13^, and photoelectrochemical cells^14^. Nevertheless, when employed as an electrode material, MoS_2_ exhibits certain inherent shortcomings. To begin with, the electrical conductivity and cyclic stability of MoS_2_ are less than optimal. Its relatively low electrical conductivity can hinder efficient electron transfer, while its cyclic stability may not meet the long-term demands of various energy storage applications. Furthermore, MoS_2_ undergoes sulfidation reactions during the charge and discharge cycles, contributing to the gradual loss of its capacity over time. This sulfidation process is one of the key factors responsible for the diminishing performance of MoS_2_-based electrodes. Lastly, the volume expansion and contraction experienced by MoS_2_ during the charge-discharge processes pose additional challenges, limiting its practical utilization in commercial applications^15^. These drawbacks highlight the need for further research and development to address these issues and optimize the performance of MoS_2_-based materials for energy storage applications. Researchers have explored various strategies to enhance the electrochemical properties of MoS_2_, aiming to mitigate its inherent limitations. These strategies encompass approaches such as extending the interlayer distance of MoS_2_, fine-tuning its nanoscale morphology^16^, leveraging carbon nanomaterials^17^ as conductive matrices, and integrating MoS_2_ with other conductive materials^5^. Among these strategies, the synergistic combination of MoS_2_ with other materials, most notably graphene (or other carbons), to create advanced composites has emerged as an exceptional method to address the deficiencies of pristine MoS_2_ and optimize its overall performance. Hybrid nanostructured materials have demonstrated a pivotal role in enhancing the performance of energy storage devices, including rechargeable batteries and supercapacitors (SCs). Moreover, it is worth noting that one of the notable methods of modification includes the exfoliation of MoS_2_, which enables the production of ultrathin MoS_2_ nanosheets with improved electrochemical properties and higher surface area, further extending its applicability in energy storage applications^18,19^.

The incorporation of carbon, either through direct synthesis or post-treatment methods, can enhance the electrical conductivity, provide mechanical support, and prevent aggregation of MoS_2_ nanosheets^20–22^. Additionally, carbon-based composites offer synergistic effects that can significantly enhance the overall performance of energy storage devices^23,24^. Energy-storage devices based on carbons exhibit excellent electrochemical performance^25–27^. Moreover, graphene or graphite furnishes enough void space to adjust the volume change during the repeated insertion or extraction of ions and fortifies the electrolyte/electrode contact area to improve the capacity of electrochemical storage devices^28^.

This study introduces an innovative approach to the synthesis and characterization of MoS_2_-carbon-based materials, which are promising for supercapacitor and ion battery applications. The novelty of the research lies in the unique synthesis process that integrates exfoliation, hydrothermal treatment, and pyrolysis to create MoS_2_-carbon hybrids with enhanced electrochemical properties. Our approach is distinguished by several key aspects. Firstly, the combination of exfoliation with hydrothermal treatment and pyrolysis represents a novel method that sets it apart from traditional synthesis techniques. This distinctive process has proven effective in optimizing the material properties. Secondly, we employed a comprehensive range of analytical techniques to thoroughly characterize the structural, compositional, and morphological properties of the synthesized materials. This extensive characterization provides a detailed understanding of how synthesis conditions influence material performance. In terms of electrochemical performance, our study demonstrates that the hydrothermally treated MoS_2_-carbon hybrids exhibit significant improvements in capacitance and charge storage efficiency. Furthermore, the materials show excellent long-term stability, with minimal performance degradation observed even after extensive testing.

## Experimental section

Materials synthesis

### Suspension preparation and exfoliation

A suspension was prepared by adding 1 g of MoS_2_ powder (the synthesis method is described in the previous report^29^), 4 g of glucose (99.5% Merck), and 80 ml of formaldehyde solution (36.5–38% in H_2_O, Merck) into a round bottom flask. The components were mixed thoroughly to obtain a homogeneous mixture.

For the exfoliation procedure, the suspension was heated at 60 °C with continuous stirring for 30 days, under a reflux condenser. Finally, the resulting MoS_2_/glucose suspension was divided into two parts for further processing. For comparison, the exfoliation was also performed for MoS_2_ without glucose.

### Synthesis of MoS_2_/G

The part of exfoliated suspension underwent slow evaporation of formalin at 60 °C. The resulting residue was then subjected to pyrolysis at 900 °C, following the same ramping and holding times as above. This material was denoted as MoS_2_/G.

### Synthesis of MoS_2_/G-H

The second part of the exfoliated suspension was transferred into a hydrothermal reactor. The hydrothermal process was carried out at 180 °C for 24 h. Subsequently, the material was filtered out and subjected to pyrolysis at 900 °C with a heating rate of 2^o^C/min and a holding time of 3 h in argon flow. This material was designated as MoS_2_/G-H.

**Fig. 1:**
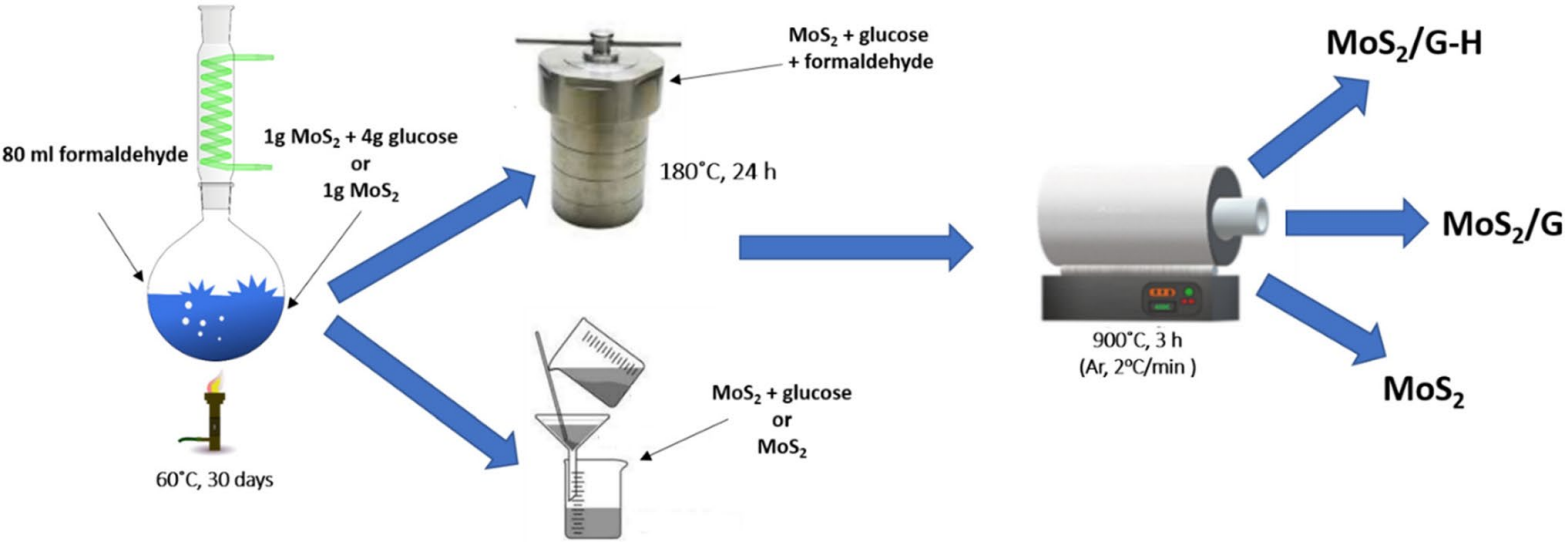
Schematic illustration for the synthesis of MoS_2_, MoS_2_/G, and MoS_2_-G/H.

The fabrication process of the materials is illustrated in Fig. 1.

#### Characterization techniques

Raman spectra were acquired using a Raman spectrometer equipped with a laser excitation source (wavelength 532 nm). The morphological features and surface morphology of the materials were examined using scanning electron microscopy (SEM) type 1430 VP, at an accelerating voltage of 30 kV from LEO Electron Microscopy Ltd with energy dispersive X-ray spectroscopy (EDX). Transmission electron microscopy (TEM) analysis was performed using a Tecnai F20 X-Twin microscope operating at an accelerating voltage of 200 kV. The TEM samples were prepared by drop-casting onto carbon-coated copper TEM grids. Before casting, the powder was dispersed in ethyl alcohol through ultrasonication to ensure a homogeneous suspension. X-ray diffraction (XRD) analysis of the materials obtained was performed using a Philips X “Pert X-ray diffractometer, with an X’Celerator Scientific detector employing Cu Kα radiation (λ = 0.15406 nm), angles ranging from 10° to 80° with step size 0.02. XPS analysis was conducted using the PHI VersaProbeII instrument (ULVAC-PHI, Chigasaki, Japan), utilizing focused, monochromatic X-ray radiation of the Al Kα line (1486.6 eV). The beam was focused to a spot of 100 µm in diameter and scanned an area of 400 µm x 400 µm on the sample surface. For each measurement point, a broad-range spectrum (0–1200 eV) was acquired with low resolution (0.5 eV), along with high-resolution spectra (0.1 eV) in the regions of the following lines’ presence: C 1s, O 1s, Mo 3d, and S 2p. Charge neutralization of the surface was employed during measurements by exposing the surface to a low-energy electron beam (1 eV) and ions (7 eV Ar^+^) to maintain a constant surface potential despite the emitted photoelectrons. The vacuum level during measurements was maintained at around 5* × *10^-9^ mbar. Spectral line fitting was performed using the PHI Multipak program (v.9.9.3) with background subtraction using the Shirley method. Additionally, the specific surface area of MoS_2_, MoS_2_/G, and MoS_2_-G/H samples was determined using the Brunauer–Emmett–Teller (BET) method.

### Supercapacitor performance measurements

#### Three-electrode configuration

The electrode materials were prepared by suspending 20 mg of the sample in 0.75 mL of distilled water, 0.2 mL of isopropanol, and 0.05 mL of 5 wt% Nafion (in lower aliphatic alcohols and water). For each material, the obtained suspension was sonicated in an ultrasonic bath for 60 min. In the next step, 20 µl of prepared mixture was placed on the polished surface of the glassy carbon electrode with a diameter of 1.5 mm (mass loading was ∼3 mg/cm^2^) and then left to dry in an oven for 120 min at 60 °C. Three-electrode configuration measurements were performed in 0.2 M K_2_SO_4_ electrolyte with Ag/AgCl (3 M KCl) and platinum mesh as a reference and a counter electrode, respectively. All the electrochemical measurements were carried out on a potentiostat/galvanostat (BioLogic VSP 2078) using cyclic voltammetry (CV) and galvanostatic charge-discharge (GCD). CV of each three-electrode setup was investigated from − 0.1 to + 0.8 V vs. Ag/AgCl (3 M KCl) with a scan rate of *v* = 50 mV s^−1^ (unless otherwise stated). GCD was performed in the same potential range at a current density of *j* = 3.2 mA cm^−2^. Moreover, EIS was performed in a frequency range between 20 kHz and 1 Hz with a voltage amplitude of 10 mV at an open circuit potential.

#### Two-electrode configuratio

To construct a symmetric supercapacitor, two flexible graphite foils (GF) were combined with the obtained material electrodes. A fiberglass separator soaked in an aqueous 0.2 M K₂SO₄ electrolyte was placed between the electrodes. The GF was coated with a mixture containing the tested materials, which was then dried at 40 °C for 6 h. The mass loading of the tested materials onto the GF was determined by measuring the weight difference of the electrode material before and after applying the mixture, using an analytical balance (RADWAG XA 82/220.4Y PLUS) with an accuracy of 0.01 mg. The mass loading was approximately 6.4 mg cm^−2^ for MoS₂, 7.2 mg cm^−2^ for MoS₂/G, and 6.8 mg cm^−2^ for MoS₂-G/H. The casing foil was sealed on three sides using a plastic foil welder, and the entire setup was securely sealed using a vacuum packing machine (CAS CVP-350/MS, Hertogenbosch). Galvanostatic charge and discharge tests (10,000 cycles) were conducted for the symmetric supercapacitors at a current density of 2 A g^−1^, with voltage range of 0 to 0.8 V. The charge-discharge measurements were also carried out for obtained materials at current densities ranging from 1 to 6 A g^-1^ over a polarization range of 0 to 0.8 V. Additionally, tests for coulombic efficiency, self-discharge, and leakage current were conducted to further evaluate the performance of the supercapacitors.

### Ion (Li^+^, Na^+^, K^+^)-battery performance measurements

The electrode material was coated on a Cu current collector (Schlenk Metallfolien, Germany) from a mixture consisting of an active material (80 wt%), binder PVDF (10 wt%) (Solef 6020, Germany), and the conductive additive (10 wt%) (Carbon Black Super P^®^, Timcal Ltd., Switzerland). The electrochemical tests were performed in a two-electrode pouch cell using the electrode material described above as a working electrode, and lithium foil (AlfaAesar, USA), sodium (AlfaAesar, USA) or potassium (Onyxmet, Poland) used as both a counter and a reference electrode. The average mass of the electrode with the diameter of 10 mm was ~ 3 mg, with a thickness of 50 μm. The electrolyte solution was 1 M LiPF_6_ in EC: DMC ratio 1:1 (LP30 Merck, Germany), 1 M NaPF_6_ in EC: DMC ratio 1:1 (Sigma-Aldrich, USA) or 0.8 M KPF_6_ (Sigma-Aldrich, USA) in EC (Alfa-Aesar, USA): DMC (Alfa-Aesar, USA) ratio 1:1. The glass fiber (Schleicher&Schüll, Germany) was used as the separator. All electrochemical experiments were performed on galvanostat/potentiostat (ATLAS 1361 MPG&T, Gdansk, Poland) within the potential range from 0.01 V to 3.0 V vs. the reference electrode. Electrochemical impedance spectroscopy (EIS) was conducted using a potentiostat equipped with a built-in impedance analyzer (BioLogic VSP 2078, Seyssinet-Pariset, France). The measurements were carried out within a frequency range of 100 kHz to 10 mHz and an amplitude of 10 mV, evaluating the electrode material in the potential window of 0.02 V to 1.5 V vs. Li/Li^+^.

## Results and discussions

**Fig. 2:**
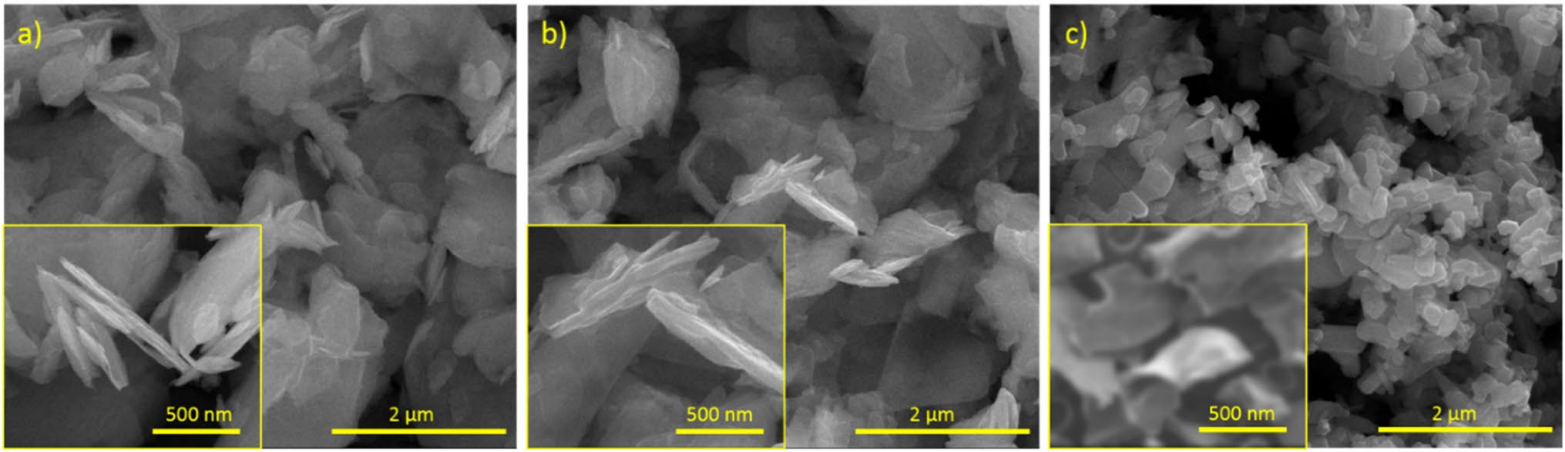
Surface SEM images of (**a**) MoS_2_, (**b**) MoS_2_/G, and (**c**) MoS_2_/G-H.

**Fig. 3:**
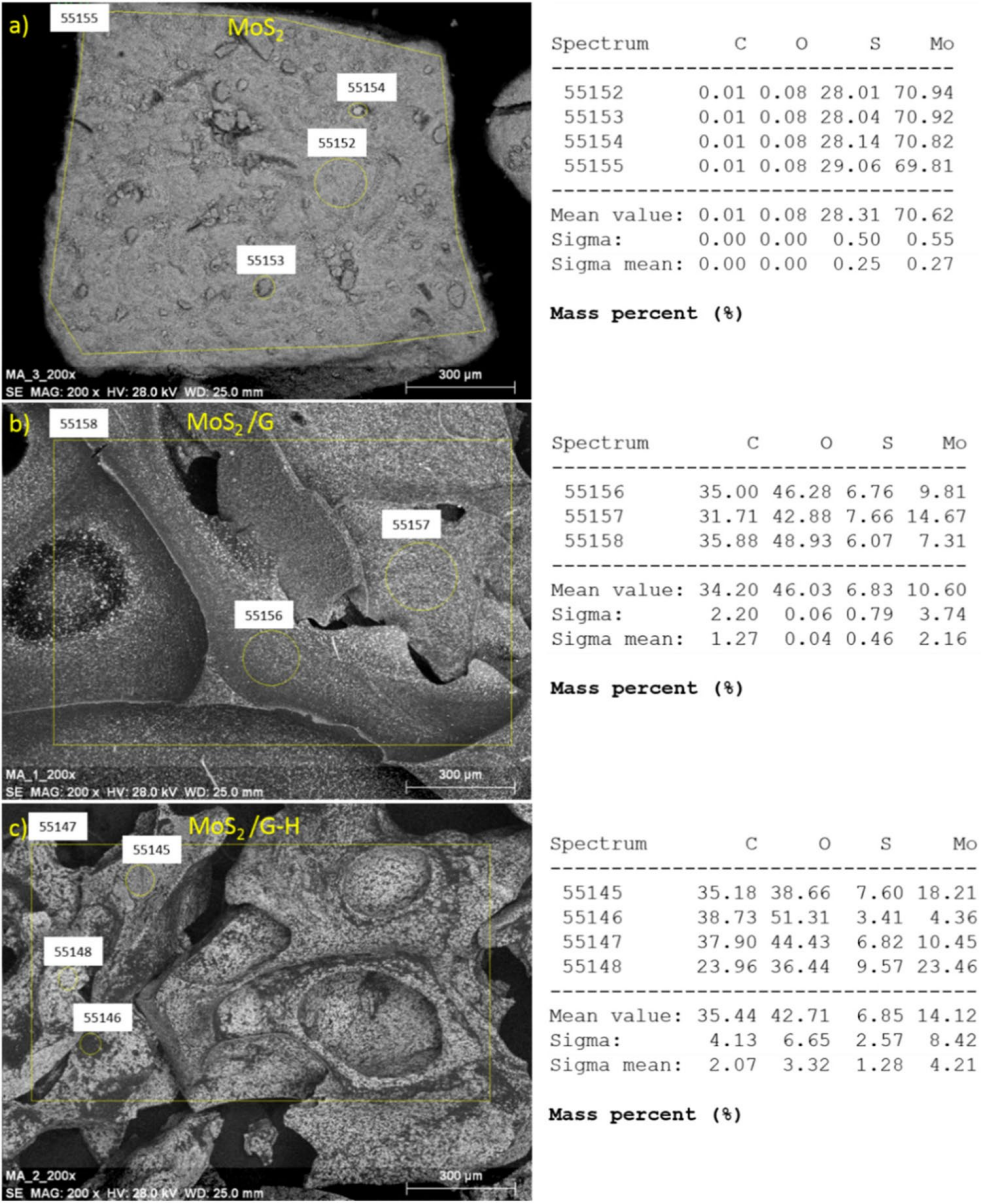
The characterization of elemental composition at various measurement sites for: (**a**) MoS_2_, (**b**) MoS_2_/G and (**c**) MoS_2_/G-H.

The SEM image of pure MoS_2_ after exfoliation (Fig. 2a) indicate that the materials exhibited a layered structure with a smooth and relatively uniform surface. The nanosheets appeared to be well-dispersed without significant aggregation. For comparison, the SEM image of MoS_2_ before the exfoliation process is presented in the Supplementary Information (see Fig. S1). The EDX analysis confirmed the presence of molybdenum (Mo) and sulfur (S) elements, consistent with the composition of MoS_2_ (Fig. 3a). SEM analysis of the MoS_2_/G material (Fig. 2b) revealed a similar morphology to the pure MoS_2_ nanosheets. The nanosheets showed good dispersibility and a smooth surface. EDX analysis demonstrated the presence of C and O elements, as expected, indicating the successful incorporation of these elements into the MoS_2_ structure (Fig. 3b). The SEM image of MoS_2_/G-H (Fig. 2c) shows that the material exhibited a modified surface morphology compared to the pure MoS_2_ nanosheets and MoS_2_/G. The addition of the hydrothermal treatment appeared to introduce some changes in the morphology, resulting in a more porous and rough surface. EDX analysis confirmed the presence of Mo, S, and additional elements corresponding to carbon (C) and oxygen (O), which can be attributed to the introduction of glucose during the synthesis process (Fig. 3c).

The obtained Energy-Dispersive X-ray Spectroscopy (EDX) results (Fig. 3) showed small variations in elemental composition depending on the measurement site for the obtained three materials. For the pure MoS_2_ material, the EDX analysis revealed the presence of sulfur (S) and molybdenum (Mo) as the primary constituents. However, in the case of the MoS_2_/G and MoS_2_/G-H materials, additional elements were detected alongside sulfur and molybdenum. These additional elements included carbon (C) and oxygen (O). The presence of carbon indicates the successful incorporation of glucose, which serves as a carbon source, into the MoS_2_ matrix. The presence of oxygen suggests the involvement of oxygen-containing functional groups or oxides in the composite materials. Thus, the EDX analysis demonstrated that the composition of the MoS_2_-based materials is influenced by the presence of glucose and the hydrothermal process. The incorporation of glucose introduces carbon and potentially oxygen into the MoS_2_ matrix, which can significantly impact the materials’ properties and potential applications. Moreover, the EDX results show that the hydrothermal step does not significantly affect the composition of the MoS_2_ with carbon.

Furthermore, EDX mapping was performed to obtain a comprehensive understanding of the elemental distribution across the synthesized MoS_2_-based materials. The mapping results provide detailed information about the spatial distribution of different elements within the materials (see Fig. S2). In the EDX mapping analysis, particular attention was given to analyzing the elemental composition of molybdenum, sulfur, and carbon within the synthesized MoS_2_-based materials. These elements play crucial roles in determining the structural and electrochemical properties of the materials.

**Fig. 4:**
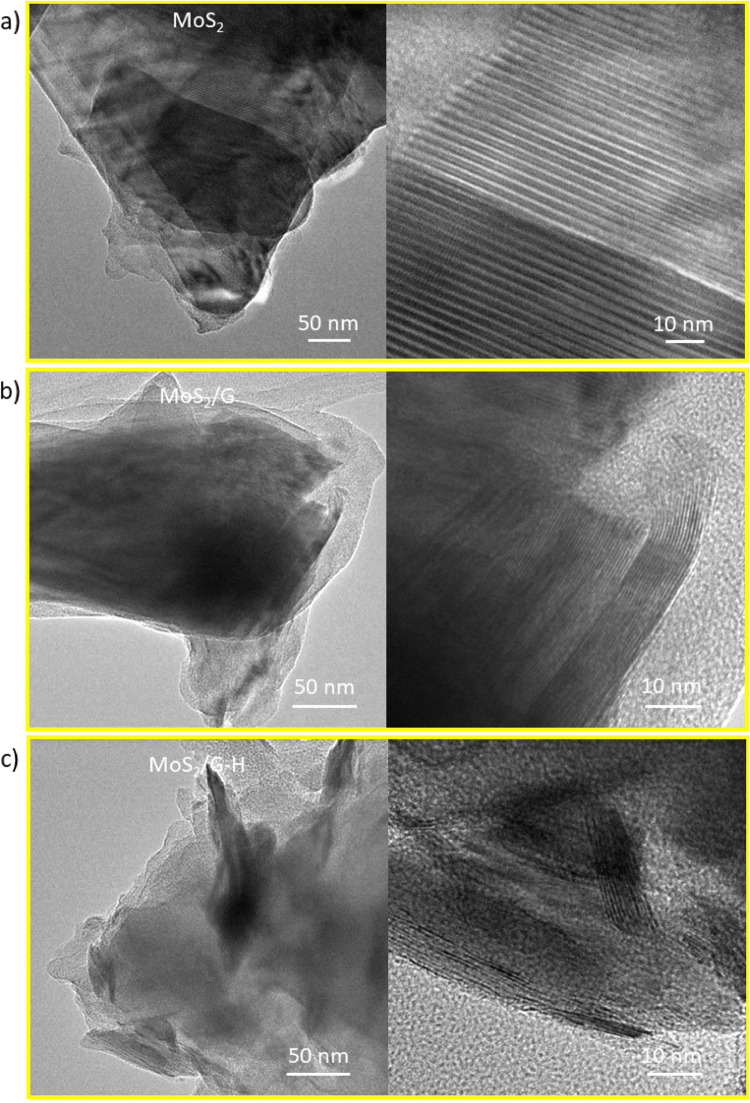
TEM images of (**a**) MoS_2_, (**b**) MoS_2_/G, and (**c**) MoS_2_/G-H with different magnifications.

Transmission electron microscopy images of the MoS_2_ sample at varying magnifications (Fig. 4a) demonstrate that the basal planes of the exfoliated molybdenum disulfide nanosheets exhibit a highly crystalline structure and confirm the uniform morphology. In contrast, the MoS_2_/G sample (Fig. 4b) and MoS_2_/G-H sample (Fig. 4c) not only reveal the crystalline MoS_2_ nanosheets, but also amorphous regions containing carbon, which originates from the carbon precursor used during the hydrothermal process and subsequent pyrolysis. These amorphous carbon regions, rich in substantial defects, are likely to introduce additional active sites, potentially enhancing charge storage capacity.

**Fig. 5:**
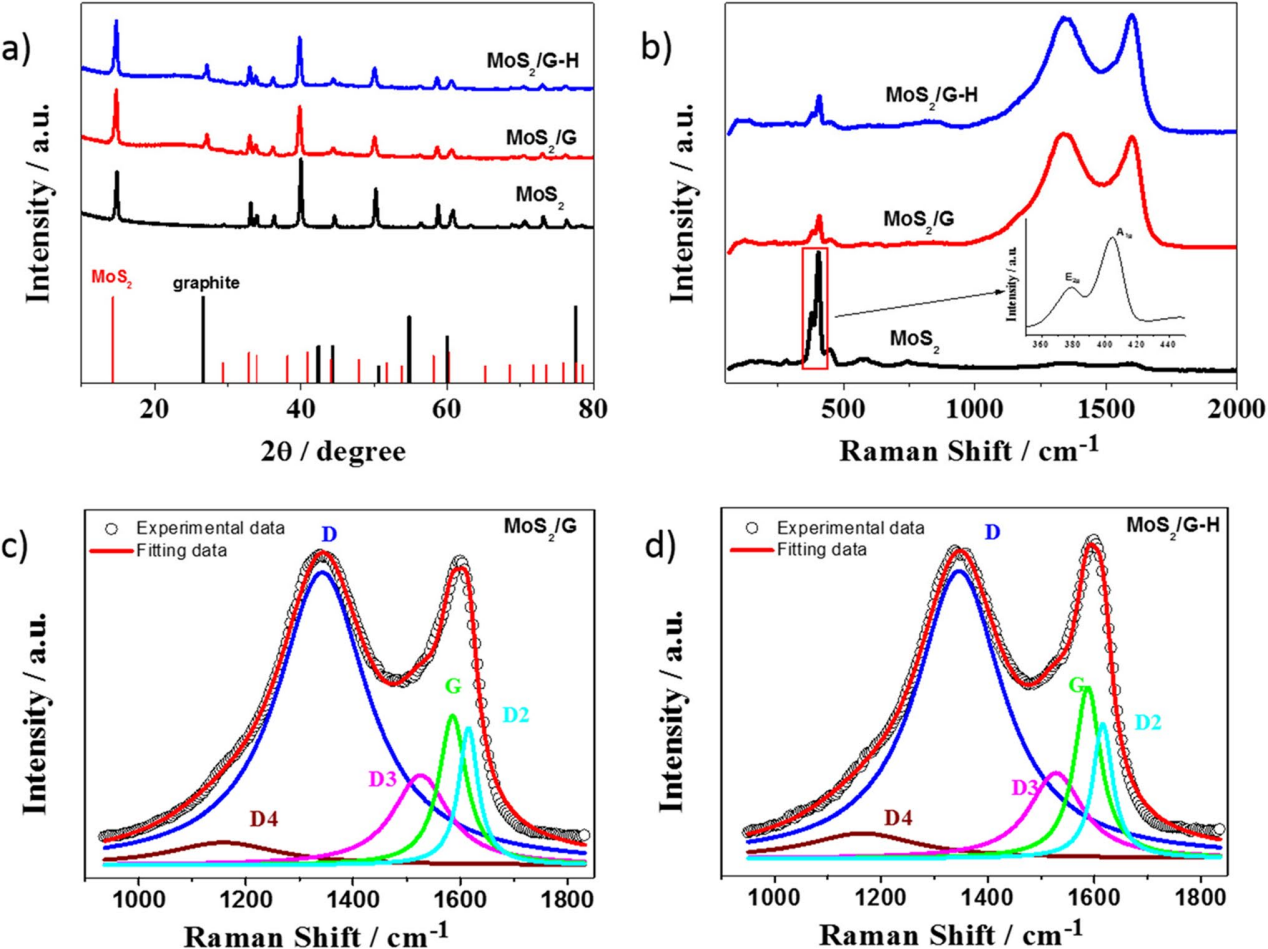
(**a**) XRD patterns and (**b**) Raman spectra for MoS_2_-based materials; Curve fit for the first-order Raman spectra for (**c)** MoS_2_/G and (**d**) MoS_2_/G-H.

X-ray diffraction (XRD) analysis was conducted to examine the crystal structure and phase composition of the synthesized MoS_2_-based materials (Fig. 5a). The XRD pattern of the pure MoS_2_ nanosheets exhibited distinct diffraction peaks at specific angles, indicating the crystalline nature of the material. The strong and sharp peaks at angles 14.8°, 39.8°, and 50.2° corresponded to the (002), (103), and (105) crystallographic planes of MoS_2_, respectively^30,31^. The observed diffraction peaks matched well with the characteristic peaks reported for the hexagonal MoS_2_ crystal structure, suggesting that the exfoliation procedure hasn’t changed the crystal structure of MoS_2_^32^. The XRD patterns of the MoS_2_/G and MoS_2_/G-H materials displayed diffraction peaks that were nearly identical to those observed for pure MoS_2_ nanosheets, indicating the preservation of the MoS_2_ crystal structure. However, in addition to the MoS_2_-related peaks, an additional weak diffraction peak was observed at around 27.2° that suggests the presence of an additional phase associated with the incorporation of carbon. Further analysis revealed that the presence of this peak is attributed to the existence of graphite in the MoS_2_/G and MoS_2_/G-H studied systems. We supposed that graphite appeared in the system during followed by pyrolysis. According to the literature, the pyrolysis procedure usually leads to changes in carbon structure resulting in the observed presence of graphite, which may be confirmed by the XRD peaks appearing at 27.2°^33^. This indicates the successful incorporation of carbon from glucose, resulting in the formation of the MoS_2_-carbon composite.

Raman spectroscopy was conducted to analyze the structural properties and vibrational modes of the synthesized MoS_2_-based materials (Fig. 5b). The Raman spectrum of pure MoS_2_ nanosheets exhibited two prominent peaks. The first one, at around 378 cm^−1^ (commonly referred to as the E^1^_2 g_ mode), corresponds to the in-plane vibration of sulfur atoms against the molybdenum atoms, whereas the second one at approximately 404 cm^−1^ (A_1g_ mode) indicates the out-of-plane vibrational motion of sulfur atoms (see the inset of Fig. 5b). These characteristic peaks confirmed the presence of the hexagonal (2 H) MoS_2_ structure. In the Raman spectra of the MoS_2_/G and MoS_2_/G-H materials, the characteristic peaks of pure MoS_2_ nanosheets were still evident. Moreover, additional peaks in the range of about 1000 to 1800 cm^−1^ correspond to the presence of carbon-related vibrations. The presence of carbon-related peaks in the Raman spectra of the MoS_2_/G and MoS_2_/G-H materials indicates the successful incorporation of carbon, and in both cases confirms the formation of MoS_2_-carbon composite.

Raman spectra after fitting for the MoS_2_/G and MoS_2_/G-H electrode materials are shown in Figs. 4d and 5c. The shape of both spectra is similar, including the position and intensities of the peak maxima. Two characteristic maxima: first at the 1350 cm^−1^ (D band), attributed to the presence of disordered graphite, and the second one (G) at 1600 cm^−1^ related to the presence of ideal graphitic lattice, can be distinguished^34^. However, the proper analysis of the spectra for the carbonaceous material in the range from 1000 cm^−1^ to 1800 cm^−1^ requires taking into account the presence of both crystalline and amorphous domains. Thus, a curve fitting was conducted, assuming Lorentzian band shapes, which is used for disordered carbons^35^. Five maxima at ~ 1170 cm^−1^ (D4), ~ 1350 cm^−1^ (D), ~ 1530 cm^−1^ (D3), ~ 1590 cm^−1^ (G) and ~ 1600 cm^−1^ (D2) were identified. The relative intensities of D and G bands are affected by the type of carbonaceous material^36^, and thus it is possible to calculate a particle size of the crystalline carbon domains, known as cluster size (*L*_a_), from the equation proposed by Ferrari and Roberts for disordered and amorphous carbons^35^:

1 $$\:\frac{I\left(D\right)}{I\left(G\right)}=0.0055\bullet\:{L}_{a}^{2}$$

The calculated cluster size is equal to 1.9 nm and 1.7 nm for MoS_2_/G and MoS_2_/G-H, respectively. This is much lower than the grain size of graphite and similar as in case of pyrolyzed lignin^37^. In summary of the Raman spectroscopy analysis, the results suggest that the carbonaceous material exhibits some resemblance to graphite-like structure, yet it also displays the presence of amorphous components.

**Fig. 6:**
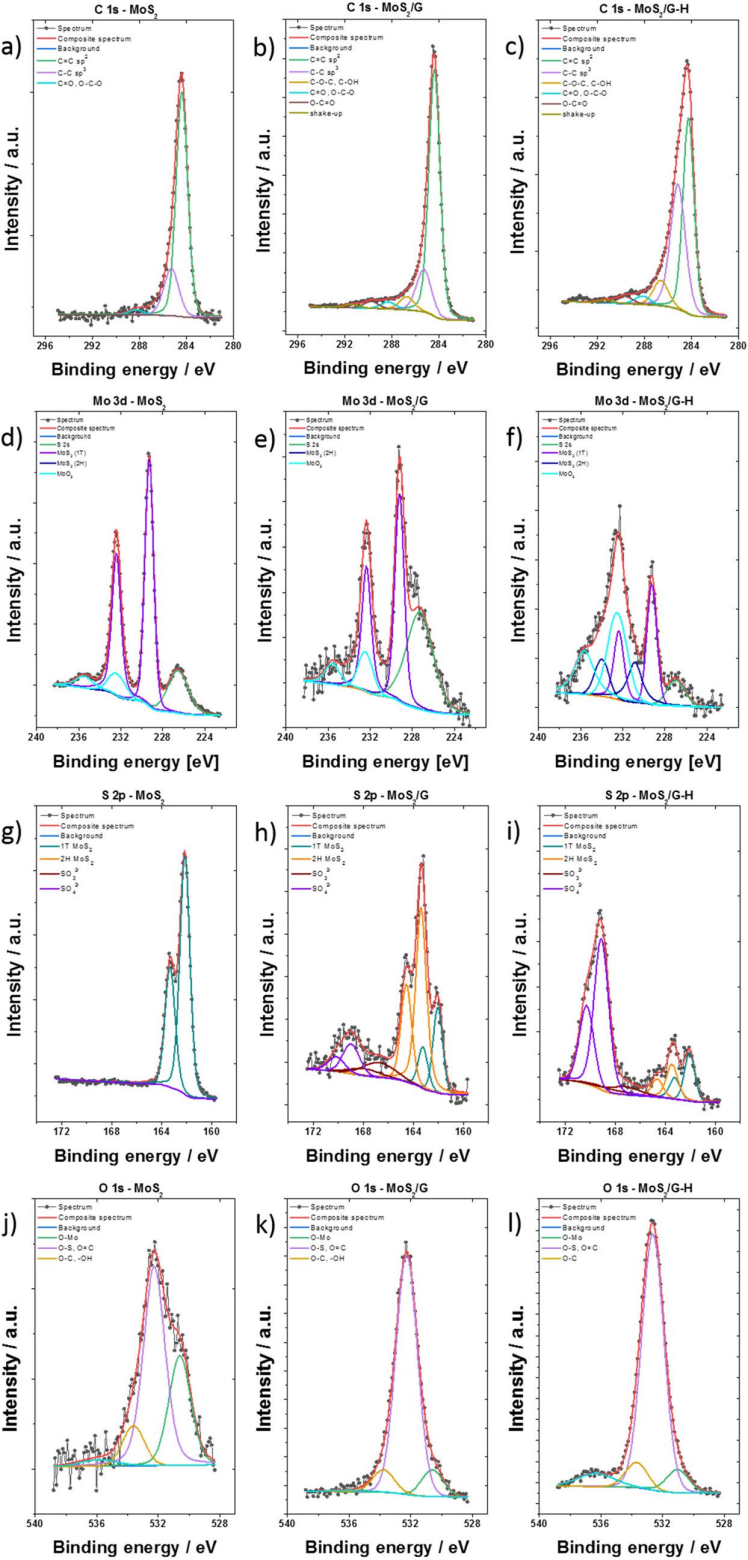
Deconvoluted XPS spectra for the MoS_2_-based materials in (**a** **b**, **c**) C 1s, (**d**, **e**, **f**) Mo 3d, (**g**, **h**, **i**) S 2p and (**j**, **k**, **l**) O 1s regions.

The X-ray Photoelectron Spectroscopy (XPS) technique was also applied to investigate the samples. The process of deconvoluting the high-resolution spectra reveals the presence of elements within diverse chemical environments. The spectrum in the C 1s region for MoS_2_/G and MoS_2_/G-H materials (Fig. 6b **and c**) was fitted with six lines, among which line 1 located at an energy of 284.4 eV represents a C = C (sp^2^) bonding^38^. Line 2 at 285.0 eV corresponds to the presence of C-C (sp^3^) bonding^38,39^. Line 3 at 286.7 eV corresponds to the presence of C-O-C and/or C-OH bonds^38,39^. Line 4 positioned at 288.3 eV corresponds to C = O and/or O-C-O bonds^38,39^. Line 5 at 289.7 eV is associated with O-C = O bonds^38^, and line 6 situated at a binding energy of 291.4 eV is linked to a shake-up excitation^40^. The “shake-up” excitation originates from sp^2^ carbon, particularly from its aromatic forms, serving as an additional parameter confirming the presence of such bonds^38,41^. In the case of MoS_2_, the presence of carbon was also observed (**see** Fig. 6a), but the signal intensity was lower than for hybrid materials. Moreover, a smaller number of lines were fitted to this spectrum.

The Mo 3d spectrum (Fig. 6d, e, f) was fitted with three doublets, characterized by a separation of d_5/2_ – d_3/2_ of 3.13 eV. The first main 3d_5/2_ line, positioned at a binding energy of 229.3 eV, indicates the presence of molybdenum sulfide in the 1T phase. The second 3d_5/2_ line, observed at a binding energy of 230.5 eV, signifies the presence of molybdenum sulfide in the 2 H phase^42^. The last 3d_5/2_ line, located at a binding energy of 232.4 eV, points to the presence of molybdenum (VI) oxide^43^. An additional line at a binding energy of around 226 eV originates from the S 2s line. Furthermore, it’s important to note that for MoS_2_, considerably sharper peaks were obtained compared to the other two materials after carbon modification. Moreover, the MoS_2_ material is primarily composed of the 1T phase (with the absence of the 2 H phase) and a minor amount of molybdenum oxide. In the MoS_2_/G material, the 1T phase was not observed, while the MoS_2_/G-H material is a mixture of molybdenum oxide, 1T phase, and 2 H phase.

The S 2p spectra (Fig. 6g, h, i) were deconvoluted into four doublets, with a p_3/2_ – p_1/2_ separation of 1.16 eV. The first main 2p_3/2_ line, positioned at a binding energy of 162.1 eV, indicates the presence of S^2^^-^ in the molybdenum sulfide of the 1T phase. The second 2p_3/2_ line, observed at a binding energy of 163.4 eV, signifies the presence of S^2^^-^ in the molybdenum sulfide of the 2 H phase. The third 2p_3/2_ line, located at a binding energy of 167.8 eV, indicates the presence of sulfite group SO_3_^2-^. The last 2p_3/2_ line, situated at a binding energy of 168.9 eV, suggests the presence of sulfur (VI) confirming the presence of SO_4_^2-^ groups^42,44^. In the case of MoS_2_, only sulfur originating from molybdenum sulfide in the 1T phase is observable. Furthermore, it’s important to note that the material subjected to hydrothermal treatment contains certain amounts of sulfates and sulfites.

The oxygen spectra (Fig. 6j, k, l) were fitted with three lines. The first line, situated at an energy of 530.7 eV, corresponds to the presence of O-Mo bonds. The second line, positioned at a binding energy of 532.4 eV, signifies the presence of both metal oxides (oxygen in non-stoichiometric oxides), organic groups like O = C, and O-S bonds. The third line, located at an energy of 533.4 eV, indicates the presence of O-C and/or -OH bonds^45^. It’s worth mentioning that the lowest oxygen content was observed for MoS_2_, which was consistent with the results obtained from Energy-Dispersive X-ray Spectroscopy.

## Electrochemical characterization

### Supercapacitor performance measurements

#### Three-electrode configuration

**Fig. 7:**
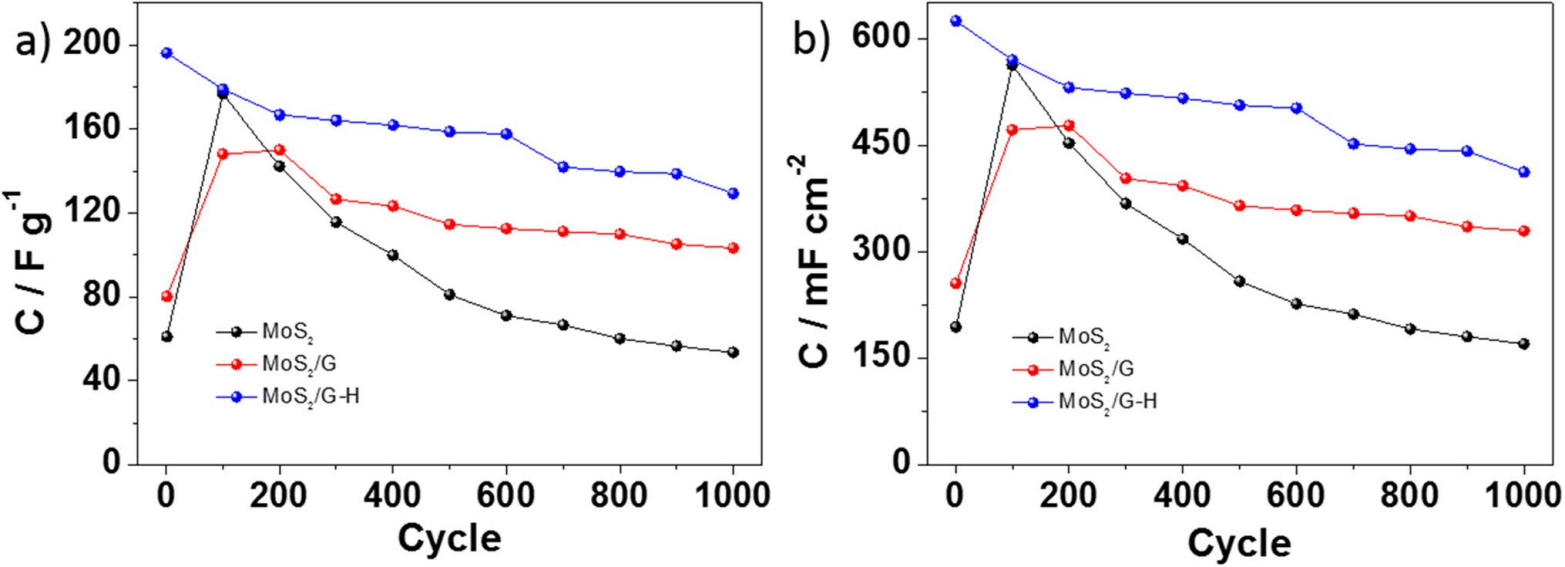
Curves of (**a**) specific (gravimetric) capacitance and (**b**) areal capacitance vs. cycle number for MoS_2_, MoS_2_/G and MoS_2_/G-H electrode materials.

For the prepared electrode materials, electrochemical measurements for supercapacitors’ application were performed in aqueous solution of 0.2M K_2_SO_4_. For both MoS_2_ and MoS_2_/G electrode materials, there was an increase in capacitance during first 100 cycles (see Fig. 7), which is frequently observed in the literature for both carbon materials^46–48^ and molybdenum sulfide^22,49^, and is associated with the activation of the electrode material and diffusion of the electrolyte into material’s structure. The results are consistent with the galvanostatic charge-discharge curves (**see** Fig. 7a and b), where charge and discharge time was prolonged at 100th cycle. In general, this is the reason for applying a “preconditioning” cycling before performing the actual galvanostatic charge-discharge tests, in order for the material to stabilize^50,51^. On the other hand, when the hydrothermal process was introduced (MoS_2_/G-H), the increase in capacitance was not observed and only a gradual decrease was noted from the 1st cycle. The hydrothermal process often induces enhanced exfoliation of the MoS_2_ nanosheets and might lead to improved accessibility of the electrolyte to the material’s inner structure, and thus preconditioning is not needed and the phenomena of the capacitance increase is not observed. Additionally, this exfoliation process may result in an increased surface area and a greater exposure of active sites available for charge storage. This potential enhancement is reflected in the BET surface area measurements, which were determined to be 6, 84, and 224 m²/g for MoS₂, MoS₂/G, and MoS₂/G-H, respectively. The nitrogen adsorption isotherms obtained at 77 K (as shown in Fig. S3) further support this observation. The shape of MoS₂/G, and MoS₂/G-H isotherms corresponds to type I according to the IUPAC classification, which indicates a microporous structure within the carbon-based materials. This microporosity suggests that the materials have a significant amount of small pores, which can contribute to improved charge storage capabilities due to the increased surface area and higher density of active sites. However, MoS₂/G-H electrode material was also characterized by the highest initial capacitance, with the 66% of capacitance retention after 1 000 cycles. The hydrothermal process could potentially lead to the formation of new surface functional groups or alter the materials surface chemistry, affecting the charge storage mechanism and the interactions with electrolyte ions. Additionally, changes in the structural characteristics induced by hydrothermal treatment could influence ion diffusion kinetics or the more effective utilization of active sites, which may positively affect the higher electrochemical capacity.

**Fig. 8:**
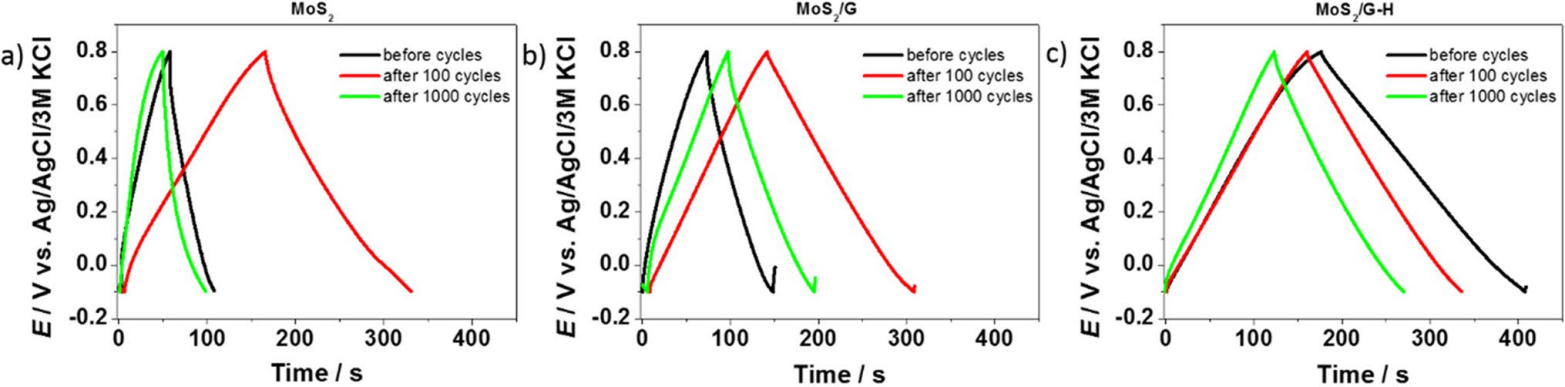
Galvanostatic charge–discharge curves recorded at 3.2 mA/cm^2^ for the (**a**) MoS_2_, (**b**) MoS_2_/G and (**c**) MoS_2_/G-H electrode materials.

**Fig. 9:**
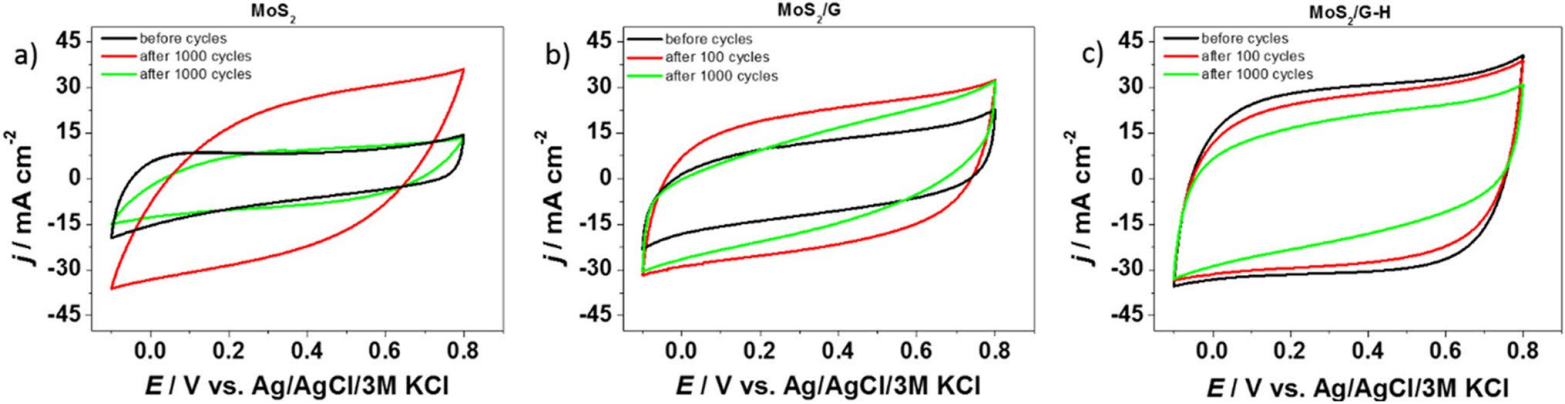
Cyclic voltammograms for the (**a**) MoS_2_, (**b**) MoS_2_/G and (**c**) MoS_2_/G-H electrode materials (a scan rate of *v* = 50 mV s^−1^).

The results are also confirmed by the galvanostatic charge-discharge curves (Fig. 8c), and for each electrode material it preserved its triangular shape. However, assuming that the initial 100 cycles are the preconditioning procedure, the MoS_2_ electrode material was characterized by the lowest capacitance retention of 30% compared to MoS_2_/G (69%) and MoS_2_/G-H (73%). It was also confirmed by cyclic voltammetry measurements (**see** Fig. 9). The electrochemical capacitance values after 1 000 cycles were 53 F g^-1^ (171 mF cm^-2^), 104 F g^-1^ (328 mF cm^-2^), 130 F g^-1^ (411 mF cm^-2^) for MoS_2_, MoS_2_/G and MoS_2_/G-H, respectively.

Figure S4 presents the electrochemical impedance spectra for the MoS₂ and carbon-based materials (MoS₂/G and MoS₂/G-H), recorded using a three-electrode setup at open circuit potential, across a frequency range of 20 kHz to 1 Hz. The absence or slight presence of a semicircle in the high-frequency region, which is typically associated with charge transfer resistance at the electrode–electrolyte interface, suggests that the materials exhibit relatively low charge transfer resistance^52^. This low resistance can be linked to the porous structure of the materials. Additionally, the reduced slope observed in the low-frequency region, compared to MoS_2_, indicates a shift from purely capacitive behavior to pseudocapacitive behavior, often signaling the occurrence of faradaic processes at the electrode surface that enhance overall capacitance. Thus, based on the impedance spectra, it can be inferred that the materials containing carbon exhibit a more capacitive character, while MoS_2_ demonstrates a diffusional character.

**Fig. 10:**
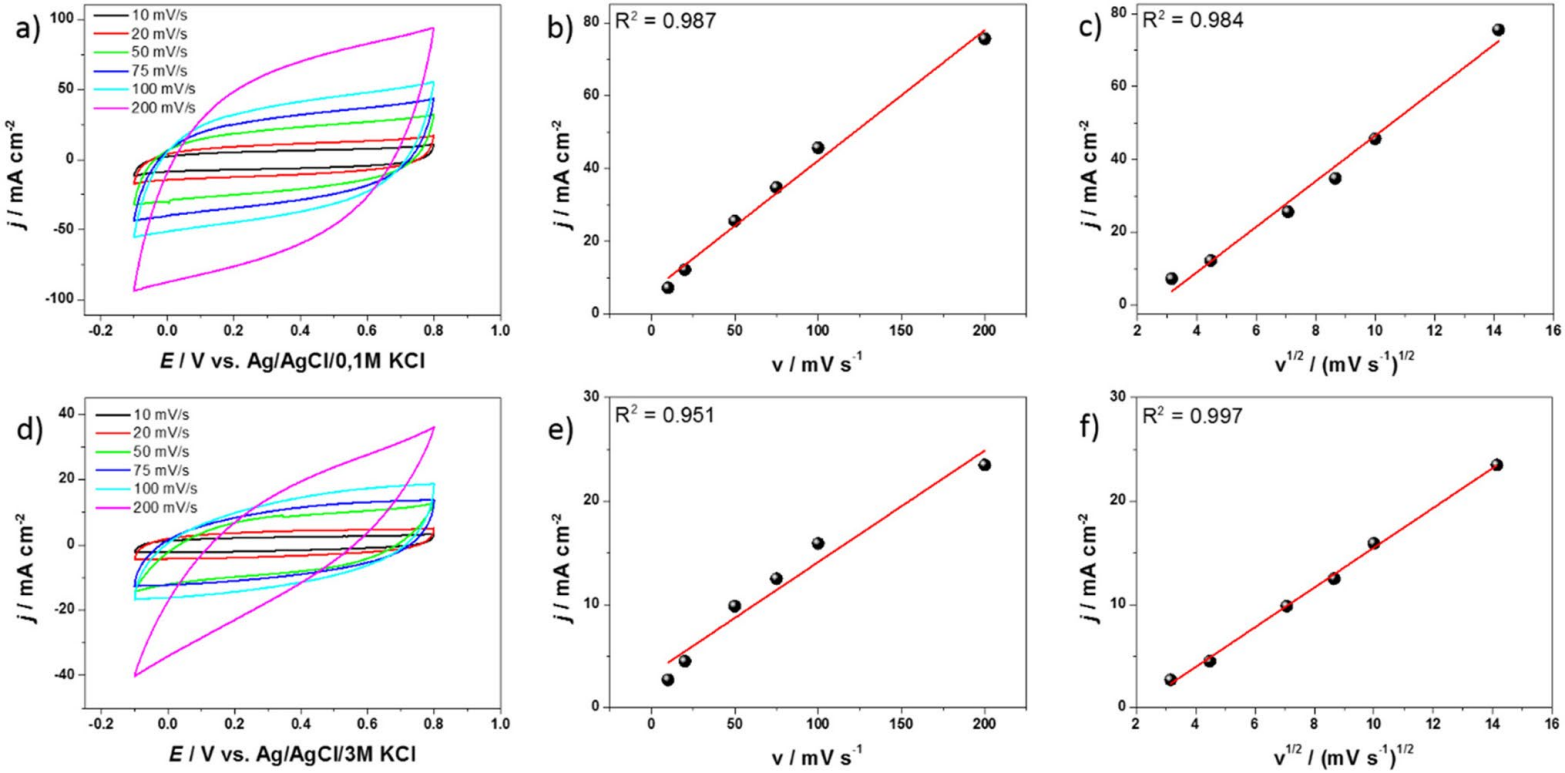
(**a**) Cyclic voltammetry of MoS_2_/G-H in 0.2 M K_2_SO_4_ at different scan rates; plots of (**b**) *j* = *f*(*v*) and (**c**) *j* = *f*(*v*^1/2^), both at E = 0.5 V.

To investigate the energy storage mechanism in the MoS_2_/G-H composite, cyclic voltammetry (CV) measurements were carried out at various scan rates. For comparison, the mechanism was also determined for MoS_2_. The CV results, shown in Fig. 9a and d, were obtained within a potential window of -0.1 to 0.8 V using scan rates of 10, 20, 50, 75, 100, and 200 mV s^−1^. To further analyze the charge storage mechanism, current density (*j*) as a function of scan rate (*v*) is plotted in Fig. 9b and e and as a function of the square root of the scan rate (*v*^1/2^) in Fig. 9c and f, both at a potential of 0.5 V. In the case of MoS_2_/G-H, the fitting results indicate that both capacitive and diffusion-controlled processes contribute to the overall charge storage in the electrode material. Given the composite nature of the MoS_2_/G-H material, this dual mechanism is expected, where each component plays a distinct role in energy storage. The carbon component, likely responsible for the capacitive behavior^53^ and enhances electrical conductivity. On the other hand, the MoS_2_ component is more associated with the diffusion-controlled mechanism^54^, where charge storage occurs through the intercalation and deintercalation of ions within its layered structure. This was confirmed by the results (see Fig. 10f), as an ideal fit of the curve was obtained from the square root of the scan rate. The combination of these two mechanisms in the composite material leads to an efficient and balanced energy storage system, leveraging the strengths of both carbon and MoS_2_.

#### Two-electrode configuration

Multiple charge-discharge cycles were conducted in a two-electrode configuration to evaluate the stability of symmetric supercapacitors based on the materials with MoS_2_ and carbon. The supercapacitors underwent an extensive cycling test of 10 000 cycles to assess their long-term stability and performance. Figure 10a presents the curves of specific capacitance vs. cycle number for the obtained electrodes, recorded at a current density of 2 A g^-1^ for the MoS_2_, MoS_2_/G and MoS_2_/G-H, respectively. In the two-electrode configuration for both MoS_2_ and MoS_2_/G based-devices, unlike in the three-electrode setup, there is no significant increase in capacitance with successive cycles. However, it is important to highlight that capacitance does continue to increase during the cycling test, particularly within the first 100 cycles. The electrodes containing carbon (MoS_2_/G and MoS_2_/G-H) demonstrated relatively good capacitance retention over 10,000 cycles. Even after this extended cycling period, these electrodes maintained almost 90% of their initial capacitance, highlighting their stability and durability. This performance can be attributed to the presence of carbon, which enhances the structural integrity of the composite and provides improved electrical conductivity, thereby reducing the overall degradation during repeated cycling. In contrast, the pure MoS_2_ electrode exhibited the lowest stability, retaining only 64% of its initial capacitance after 10 000 cycles. MoS_2_ alone is more susceptible to performance deterioration during long-term cycling without the added mechanical support and conductivity the carbon component provides. This comparison underscores the importance of carbon in enhancing the long-term stability of electrode materials. Table 1 compares the capacitance values from this study with those reported in the literature, including two-electrode configurations (MoS_2_-based electrodes). The capacitance values are presented either in Farads per gram (F/g) or Farads per square centimeter (F/cm²), depending on the specific measurement method used. As shown in Fig. 10b, the coulombic efficiency for all materials is nearly 100% after 10 000 cycles. However, during the initial cycles (up to around 400 cycles, as highlighted in the inset of Fig. 10b), the coulombic efficiency exceeds 100%, particularly for the MoS_2_ and MoS_2_/G materials. This phenomenon could be attributed to the initial increase in capacitance observed for these two materials during the early stages of cycling. This increase suggests that the materials are undergoing an activation process, where the electrode structure gradually becomes more accessible to the electrolyte, enhancing charge storage capacity. The activation might involve the gradual exfoliation of MoS_2_ layers or the formation of additional electrochemically active sites, which contribute to the apparent over performance in coulombic efficiency during the initial cycles.

**Table 1:** Comparison of the specific capacitance values for the synthesized materials and MoS_2_-based symmetric devices described in the literature.

Electrode material	Specific capacity	Current density	Ref.
MoS_2_	28 F/g	2.0 A/g	This work
MoS_2_/G	56 F/g	2.0 A/g	This work
MoS_2_/G-H	81 F/g	2.0 A/g	This work
MoS_2_@CF	495 mF/cm^2^	6 mA/cm^2^	^55^
2D MoS_2_/Sb_2_S_3_	360 F/g	3 A/g	^56^
MoS_2_ nanosheets	129 F/g	1 A/g	^57^
MoS_2_ nanostructure	106 F/g	-	^58^
MoS_2_/GF//AEG	59 F/g	1 A/g	^59^
MoS_2_ nanoworms	138 F/g	1 A/g	^60^
MoS_2_/RCF composite	225 F/g	0.5 A/g	^61^

**Fig. 11:**
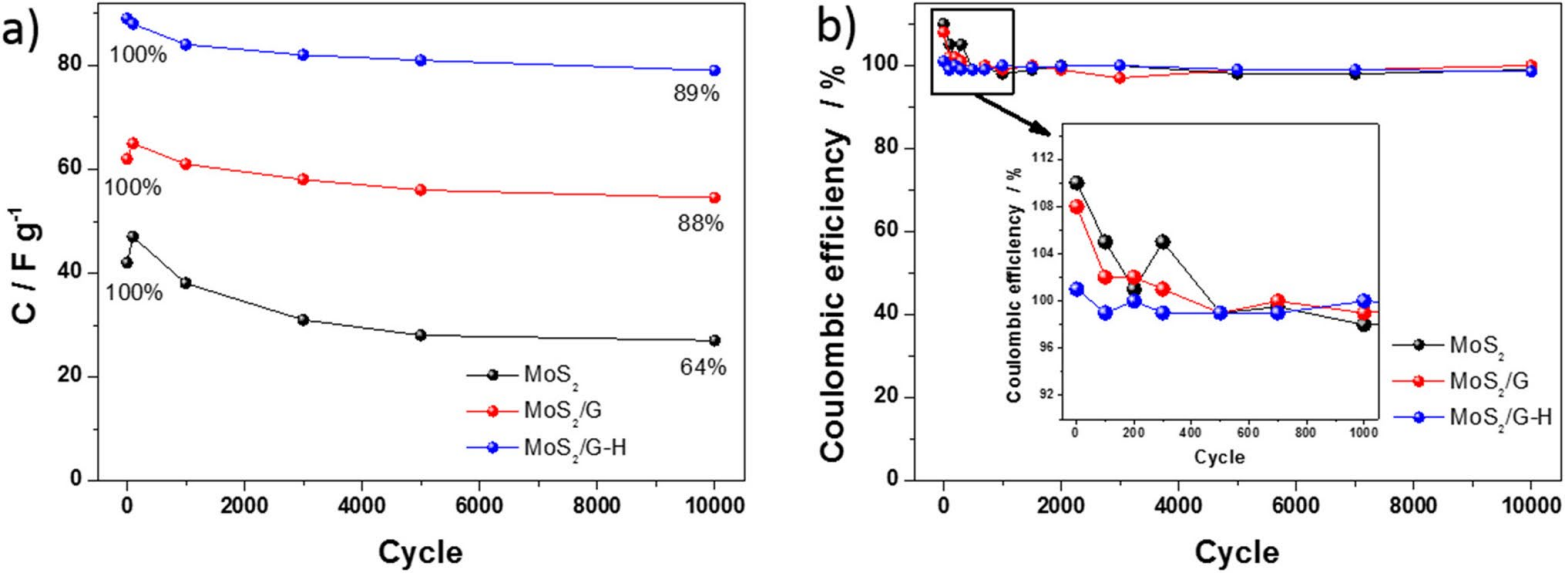
(**a**) Capacitance and (**b**) coulombic efficiency for the MoS_2_, MoS_2_/G and MoS_2_/G-H-based symmetric devices during 10 000 GCD cycles.

OCV decay is a crucial parameter for assessing the self-discharge behavior of a supercapacitor. Figure 11a illustrate the OCV decay profiles for the MoS_2_, MoS_2_/G, and MoS_2_/G-H supercapacitors, respectively. Prior to measuring the OCV decay, the cells underwent charging and discharging at a current density of 2 A g^−1^ for 100 cycles. Afterward, a float voltage of 1 V was applied for 0.5 h, and the open-circuit voltages (OCV) were monitored for 1 h. Among the materials tested, the pure MoS_2_ electrode exhibited the most significant OCV decay, with the voltage dropping from 1 V to 0.2 V within the first 30 min. This pronounced decay suggests a higher rate of self-discharge, which could be attributed to the lack of conductive carbon, resulting in less stable charge retention. In contrast, the incorporation of carbon into the electrode material positively influenced the OCV retention. For the MoS_2_/G composite, the OCV after 30 min was 0.36 V, indicating improved stability compared to pure MoS_2_. The MoS_2_/G-H composite showed even better performance, with the OCV remaining at 0.63 V after the same period. This improvement highlights the beneficial role of carbon in enhancing the material’s ability to maintain its charge. The presence of carbon increases conductivity, stability, and specific surface area, which likely contributes to the significantly longer self-discharge time compared to MoS_2_.

After the self-discharge tests, the leakage current for all the electrodes was measured, as shown in Fig. 11b. The measurement was conducted at a constant voltage of 1 V over 3600 s. A rapid decrease in current was observed on all electrodes within the first few minutes of the measurement. However, the materials with added carbon exhibited the fastest drop in current. After about 800 s, the current stabilized and remained nearly constant until the end of the measurement, with values below 10 µA for each material. The faster decrease in current observed for the carbon-containing materials can be attributed to the enhanced conductivity and higher surface area provided by the carbon component. The increased conductivity allows for quicker charge redistribution within the electrode material, leading to a more rapid initial current drop. The high surface area of the carbon also facilitates faster ion mobility and charge transfer processes at the electrode-electrolyte interface, resulting in a swift reduction in current during the early stages of the measurement. The carbon material may also serve as a protective layer, preventing undesirable and irreversible reactions in the MoS_2_ component. This protective role could contribute to the stability of the leakage current. The carbon layer helps shield MoS_2_ from direct exposure to the electrolyte, reducing side reactions that could otherwise increase the leakage current over time. As a result, the materials containing carbon maintain a more stable and lower leakage current throughout the measurement, further emphasizing the role of carbon in enhancing both the performance and longevity of the electrode.

**Fig. 12:**
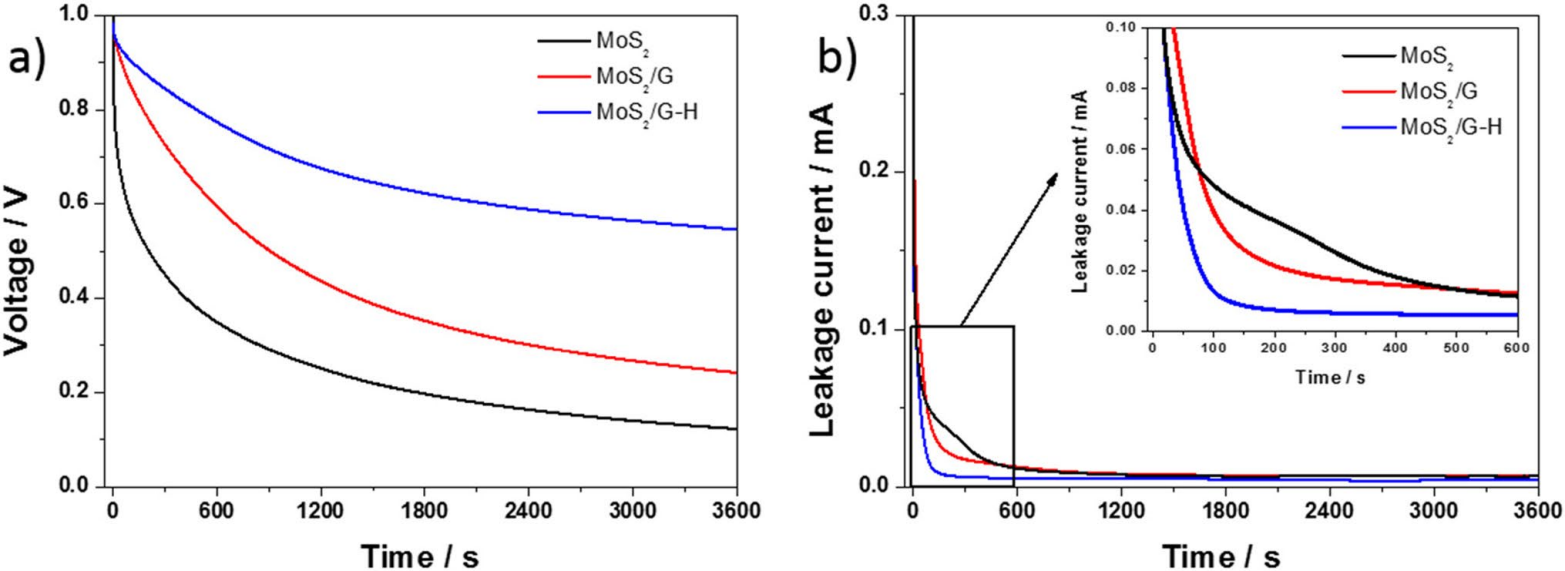
(**a**) Self-discharge plot and (**b**) leakage current curves (at a voltage of 1V)for the MoS_2_, MoS_2_/G and MoS_2_/G-H symmetric devices.

**Fig. 13:**
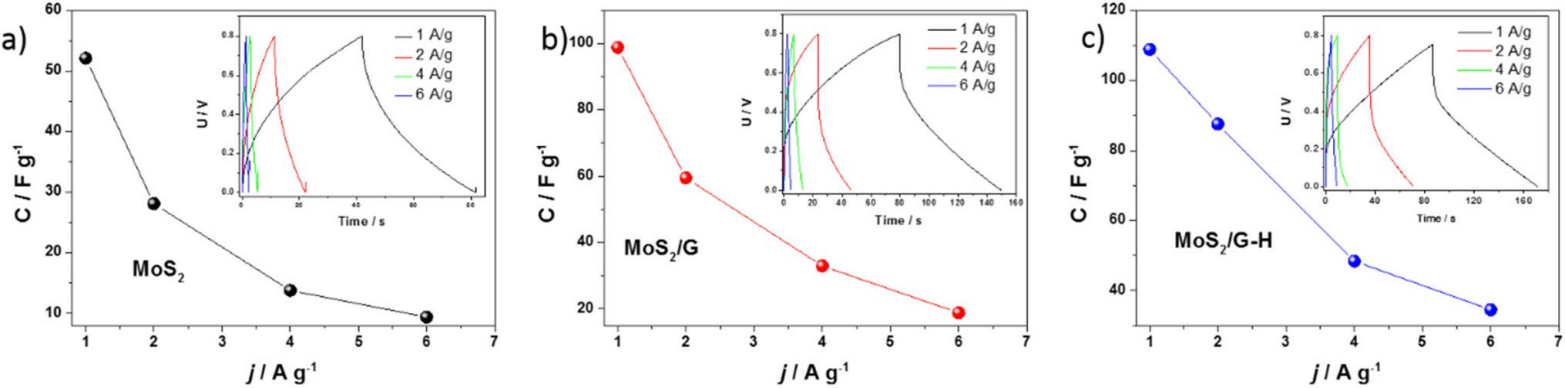
Dependence of the specific capacitance of the (**a**) MoS_2_, (**b**) MoS_2_/G and (**c**) MoS_2_/G-H based symmetric devices on the current density. Inset: Charging-discharging curves recorded at different density.

Charge–discharge measurements were performed at varying current densities for the MoS_2_, MoS_2_/G, and MoS_2_/G-H-based supercapacitors (after stability tests). The charge–discharge curves for all the materials exhibited a shape that was close to triangular, although not perfectly so, as illustrated in the insets of Fig. 13a, b, and c. This near-triangular profile is still a positive indicator for supercapacitor applications. It suggests that the materials can store and release energy relatively efficiently, but the deviation from a perfect triangle may be due to factors such as internal resistance, electrode polarization, or the inherent pseudocapacitive behavior of the materials. These factors can cause a slight distortion in the ideal triangular shape, especially at higher current densities, where rapid charging and discharging can intensify such effects. Nonetheless, the overall shape still reflects a good balance between energy storage and release, making these materials promising candidates for high-performance supercapacitors. As the charging/discharging current increases for each material, lower capacitance values are observed, as shown in the graphs in Fig. 12. This occurs because higher current densities reduce the time available for ions to migrate and fully interact with the electrode material. As a result, the electrode’s active sites are not utilized to their full capacity, leading to decreased charge storage. Additionally, at higher currents, the increased resistance and polarization effects can further contribute to the reduction in capacitance.

### Ion (Li^+^, Na^+^, K^+^)-battery performance measurements

**Fig. 14:**
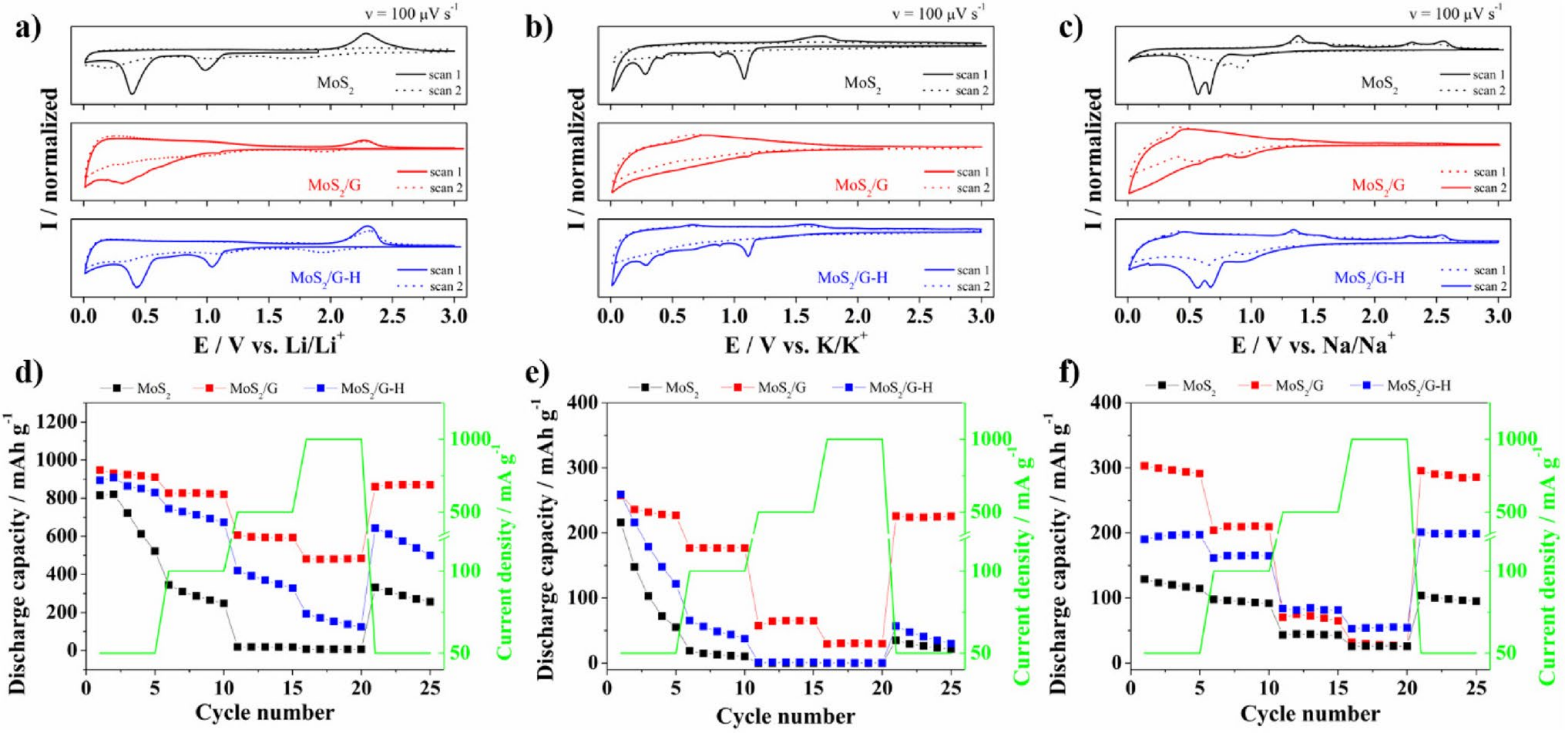
CV curves recorded for MoS_2_-based electrode materials in electrolyte containing (**a**) Li^+^, (**b**) K^+^ and (**c**) Na^+^ ions in the voltage range 0.01–3.0 V at a sweep rate of 100 µV/s. The charge/discharge cycle performance of studied electrode materials in electrolyte containing (**d**) lithium ions, (**e**) potassium ions and (**f**) sodium ions at different current densities.

Additionally, the obtained materials were investigated to evaluate their charge storage properties as a negative electrode material for lithium-, sodium- and potassium-ion batteries. Figure 14a-c shows CV curves (1st and 2nd cycle) recorded for MoS_2_, MoS_2_/G, and MoS_2_/G-H electrode materials in different electrolytes. One may see that the shape of the 1st cycle for Li^+^ (Fig. 13a) and K^+^ (Fig. 14b) differs significantly from the shape of the CV curve for the 2nd cycle. In the first scan, for the MoS_2_ and MoS_2_/G-H electrodes, there are cathodic maxima at ~ 1.0 V and 1.1 V, respectively, which are related to ion intercalation into MoS_2_ structure with the formation of Li_x_MoS_2_ or K_x_MoS_2_. Further reduction of Li_x_MoS_2_ (or K_x_MoS_2_) leads to its decomposition into Mo and Li_2_S (or K_2_S), coupled with solid electrolyte interphase (SEI) formation^62^. In the case of MoS_2_/G, the CV shape exhibits a broad signal starting at ~ 0.9 V, with a maximum at ~ 0.3 V and it may be assumed that this broad peak is associated with a simultaneous intercalation of Li^+^ ions into both MoS_2_ and carbon matrix phase. The presence of carbon matrix may hinder or diminish the response originating from the reaction between Li^+^ and MoS_2_. However, for each of the materials in the Li-half cell (**see** Fig. 14a), there is an anodic maximum at ~ 2.3 V, attributed to ion extraction and oxidation of Mo to MoS_2_^63^. It is noteworthy, that only for the MoS_2_ electrode material, the maximum is not preserved in the second scan, whereas for materials investigated in potassium-based salt, the position of the anodic maximum is shifted towards 1.7 V.

When sodium-based electrolyte was used, the CV shape recorded for the MoS_2_ and MoS_2_/G-H electrode materials are also similar. However, for all materials, one may see a small broad peak at 0.9 V, which is related to sodium intercalation into MoS_2_ with subsequent Na_x_MoS_2_ formation. The main difference in CV shape may be distinguished at the potential below 0.8 V, where two cathodic maxima at 0.7 V and 0.6 V for MoS_2_ and MoS_2_/G-H electrode materials are visible. These maxima may be attributed to the two reactions: (1) Na_x_MoS_2_ decomposition into Mo and Na_2_S (II) and (2) SEI formation^64^. It is worth noting that in the second cycle, the cathodic maximum at 0.9 V is visible regardless of the studied material, indicating that sodium intercalation into MoS_2_-based materials is a reversible process. Moreover, in the second cycle, another current maximum at ~ 0.65 V for MoS_2_/G and MoS_2_/G-H, and at ~ 0.77 V for MoS_2_ was recorded, also confirming the reversibility of the reaction between sodium ions and MoS_2_-based electrode materials. During the oxidation process, four redox maxima can be distinguished for MoS_2_ and MoS_2_/G-H at 1.3 V, broad plateau at 1.5 V, 2.3 V, and 2.6 V. The signals at 1.3 V and 1.5 V are attributed to Mo and Na_2_S oxidation to Na_x_MoS_2_, coupled with Na^+^ extraction from the carbon matrix, whereas the maxima at higher potentials are due to oxidation of Na_x_MoS_2_ with MoS_2_ formation.

The rate capability of the studied MoS_2_-based materials in LiPF_6_, NaPF_6_, and KPF_6_ is presented in Fig. 14(d-f). The common feature regardless of the electrolyte is that MoS_2_/G electrode material was characterized by the most promising electrochemical performance, whereas the discharge capacity and rate capability values were the lowest for the MoS_2_ electrode material. The specific discharge capacity after the 25th cycle at 50 mA g^−1^ for MoS_2_/G was 870 mAh g^−1^, 225 mAh g^−1^_,_ and 285 mAh g^−1^ in Li-, K- and Na-based electrolytes, respectively, whereas the capacity retention was 92%, 87% and 94% in each case, indicating that MoS_2_/G may be applied as an anode material with all studied alkali metals. Furthermore, MoS_2_/G-H turned out to be suitable as an anode material for LIBs and SIBs, showing considerably high capacity and stability, respectively. However, in the case of KIBs, only MoS_2_/G exhibited good electrochemical stability, but only at a current density of up to 100 mA g^−1^. In general, with the increase of current density, the drop in the discharge capacity is observed. It is known that it may be caused by many factors such as diffusion limitations, heat generation, electrode degradation, and overpotential effects^65,66^. Nevertheless, the MoS_2_/G electrode material was characterized by relatively good electrochemical performance in Li-based electrolyte, with a specific capacity of 341 mAh g^−1^ at 1 A g^−1^ after 800 cycles, with the capacity fade of 39% (see, Fig. S5). This is similar to what was already reported for V-doped MoS_2_^67^. In our case, the modification of undoped MoS_2_ with carbon led to the formation of Li_x_MoS_2_, followed by the formation of Mo with further incorporation of lithium ions, and ensured reversible recovery of MoS_2_^68,69^. The XPS results showed that pyrolysis of the MoS_2_/G-H material led to the formation of various MoO_x_ compounds and oxidation of S^2^^-^ to S(VI). The presence of MoO_x_ and SO_4_^2-^ may hinder lithium insertion into MoS_2_, and affect the electrochemical stability of MoS_2_/G-H. Additionally, the MoS_2_/G material is more rich in the 2 H phase than both MoS_2_ and MoS_2_/G-H. Our recent studies evidenced that crystal orientation plays a crucial role in energy storage mechanism^70^. Thus, it may be concluded that the presence of the 2 H phase in MoS_2_/G enables lithium ions to be inserted into the material much more easily than into the 1T phase. As a result, the MoS_2_/G material exhibits the best electrochemical performance among the studied materials. The presence of the 2 H phase of MoS_2_ embedded into the carbonaceous phase limits the utilization of MoS_2_ as a host material for lithium ions.

Unfortunately, the results obtained for SIBs and KIBs are not as attractive as for LIBs, and only MoS_2_/G material exhibited some potential usage. The utilization of both MoS_2_/G and MoS_2_/G-H for SIBs may be due to the presence of an amorphous hard carbon phase that is known to be suitable for sodium storage^71^. However, one may find much more promising values for MoS_2_ doped with heteroatoms^72–74^ and it is worth considering the doping before further modification with a carbonaceous matrix. Nevertheless, the MoS_2_/G with the presence of the MoS_2_ 2 H phase may be considered as promising anode material for lithium or sodium battery applications.

Additionally, SEM (Fig. S8) images and XRD (Fig. S9) measurements were conducted after stability tests on the materials used in Li-ion batteries. As observed in the SEM images, there is no significant difference in the morphology of the layers deposited on the Cu foil after electrochemical measurements. This suggests that the structural integrity of the electrode material remains largely intact during cycling, which is crucial for maintaining long-term performance. However, the XRD measurements revealed certain changes in the material layers after the electrochemical tests. It appears that the inorganic component, MoS_2_, undergoes structural modifications. This could be attributed to the insertion of lithium ions into the MoS_2_ material during cycling, which likely disrupts its crystalline structure. As a result, the material may transition to an amorphous state, explaining the absence of certain peaks, particularly at angles 14.5°, 58.2°, and 60.3°. The loss of these characteristic peaks suggests that lithium intercalation leads to significant alterations in MoS_2_ crystalline order, potentially impacting its performance and stability over time.

Due to the fact that the best battery performance was obtained for MoS_2_/G electrode material in the presence of lithium ions, electrochemical impedance spectroscopy measurements were performed for this sample to calculate the Li-ion diffusion coefficient at different potentials. Figure 7 presents 3D Nyquist plots of MoS_2_-based electrode materials at various potential values, illustrating that the shape of the plots changes depending on the material and applied potential. In contrast, the plot for MoS_2_ remains relatively unchanged across the range of potentials. However, for the MoS_2_/G and MoS_2_/G-H electrodes, a noticeable change in the shape of the Nyquist with varying voltage can be observed. As the potential decreases, a semicircle begins to form, indicating the emergence of charge transfer resistance during the insertion of lithium ions from the electrolyte into the electrode material. A comparison of the Li-ion diffusion coefficients is given in Fig. 8, showing that the value of D_Li+_ value changes with potential and is two order of magnitude lower for pure MoS_2_ (~ 5·10^–12^ cm^2^/s) compared to MoS_2_ with a carbon phase (~ 5·10^–10^ cm^2^/s). These values are approximately 10^8^ times higher than those for unannealed and annealed MoS_2_/C materials^75^. This highlights the role of carbon matrix in significantly enhancing lithium-ion transport within the electrode material.

## Conclusions

This study details the synthesis and characterization of MoS2-based materials for use in energy storage devices like supercapacitors and ion batteries. The materials, synthesized through exfoliation, hydrothermal treatment, and pyrolysis, were analyzed using techniques such as Raman spectroscopy, XRD, XPS, SEM, and EDX. Structural analysis confirmed the preservation of MoS2’s hexagonal crystal structure, while the addition of glucose introduced carbon, forming MoS2-carbon composites. XRD and Raman spectroscopy revealed the presence of graphite, and XPS confirmed the coexistence of molybdenum sulfide and oxide phases. Comprehensive electrochemical tests, including cyclic voltammetry (CV), electrochemical impedance spectroscopy (EIS), and galvanostatic charge-discharge measurements, demonstrated that the MoS_2_/G-H material, produced via hydrothermal methods, showed enhanced stability and capacitance in supercapacitor. MoS_2_/G performed well as a negative electrode in lithium-ion batteries, with good discharge capacities and rate capabilities. Overall, the study highlights the potential of MoS_2_-based materials for energy storage, offering insights into their tailored properties for improved performance in emerging technologies.

## Electronic supplementary material

Below is the link to the electronic supplementary material.

Supplementary Material 1

## Data Availability

The datasets generated and/or analyzed during the current study are available in the BRIDGE OF KNOWLEDGE repository (https://mostwiedzy.pl/pl/open-research-data/xrd-for-mos2-carbon-based-materials,730095711711579-0, 10.34808/xm0j-5202).
