# Supplementary material for: Synthesis and characterization of MoS2-carbon based materials for enhanced energy storage applications
Source: Sci Rep. 2024 Oct 30;14:26128. doi: 10.1038/s41598-024-77545-w (PMC11525990; doi:10.1038/s41598-024-77545-w)
Supplement: Supplementary file 1 — Supplementary Material 1 [file 41598_2024_77545_MOESM1_ESM.docx]

Supplementary Materials

Synthesis and Characterization of MoS_2_-Carbon Based Materials for Enhanced Energy Storage Applications

M. Szkoda^1,2*^, A. Ilnicka^3^, K. Trzciński^1,2^, Z. Zarach^1^, D. Roda^1^, A. P. Nowak^1,2^

^1^Faculty of Chemistry, Department of Chemistry and Technology of Functional Materials, Gdańsk University of Technology, Narutowicza 11/12, 80-233 Gdańsk, Poland

^2^Advanced Materials Center, Gdańsk University of Technology, Narutowicza 11/12, 80-233 Gdańsk, Poland

^3^Faculty of Chemistry, Nicolaus Copernicus University in Torun, Gagarina 7, 87-100 Toruń, Poland

mariusz.szkoda1@pg.edu.pl

**
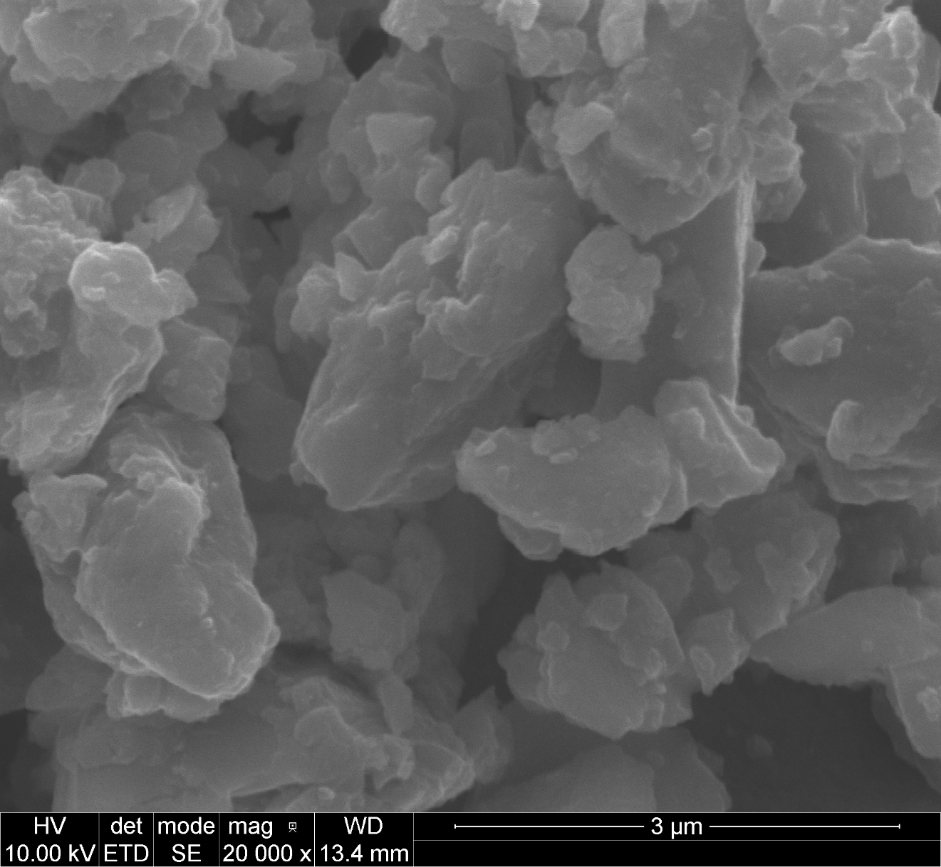
**

**Fig. S1.** SEM image of MoS_2_ before the exfoliation process.


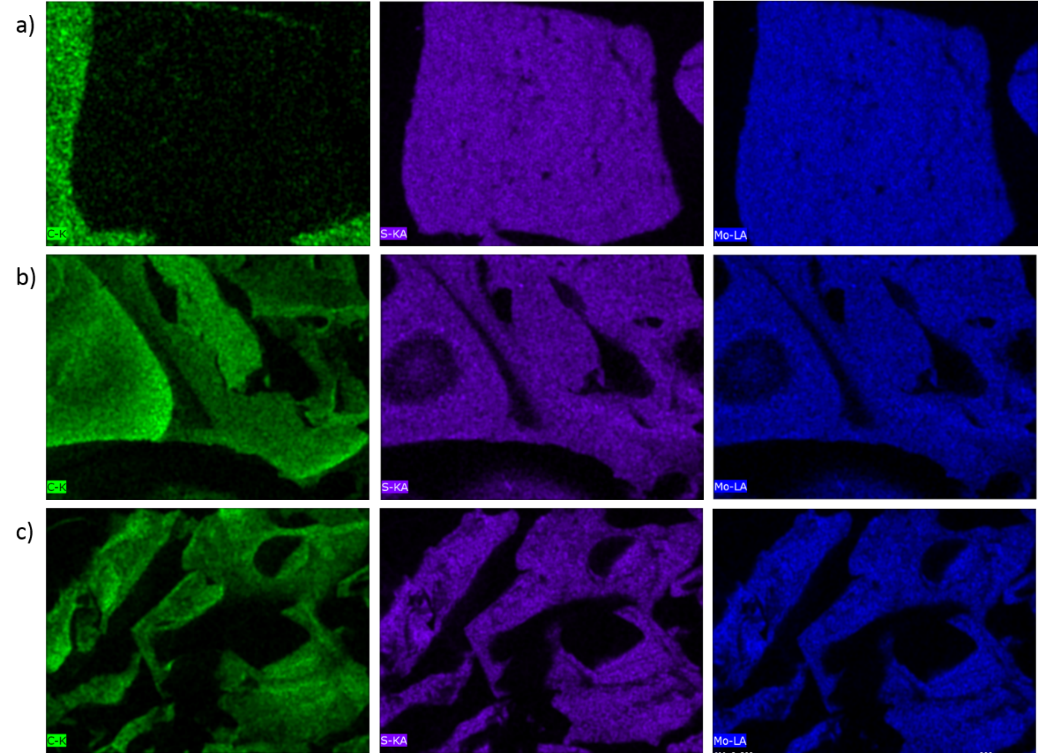


**Fig. S2.** Elemental mapping for the obtained materials: a) MoS_2_, b) MoS_2_/G, and c) MoS_2_/G-H.

**

**

**Fig. S3.** N_2_ adsorption isotherms of MoS_2_, MoS_2_/G, and MoS_2_-G/H.





**Fig. S4.** Electrochemical impedance analysis of the obtained electrode materials in a three-electrode configuration at an open circuit potential (in a frequency range of 20 kHz to 1 Hz).





**Fig. S5.** Electrochemical capacity depending on the charge/discharge cycle (800 cycles) for MoS_2_/G (Li-based electrolyte).


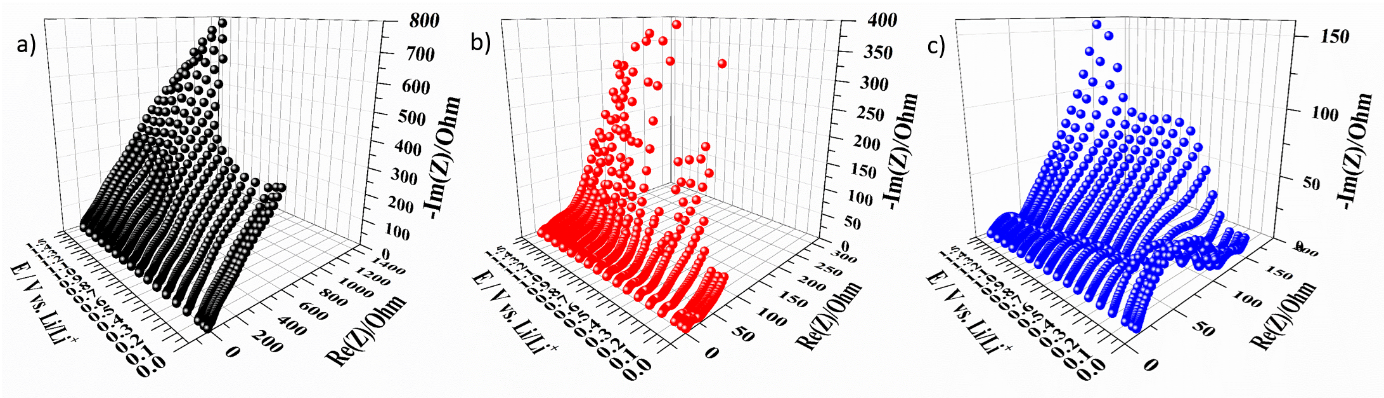


**Fig. S6.** 3D Nyquist plots of a) MoS_2_, b) MoS_2_/G, and c) MoS_2_/G-H electrode materials at different voltages.


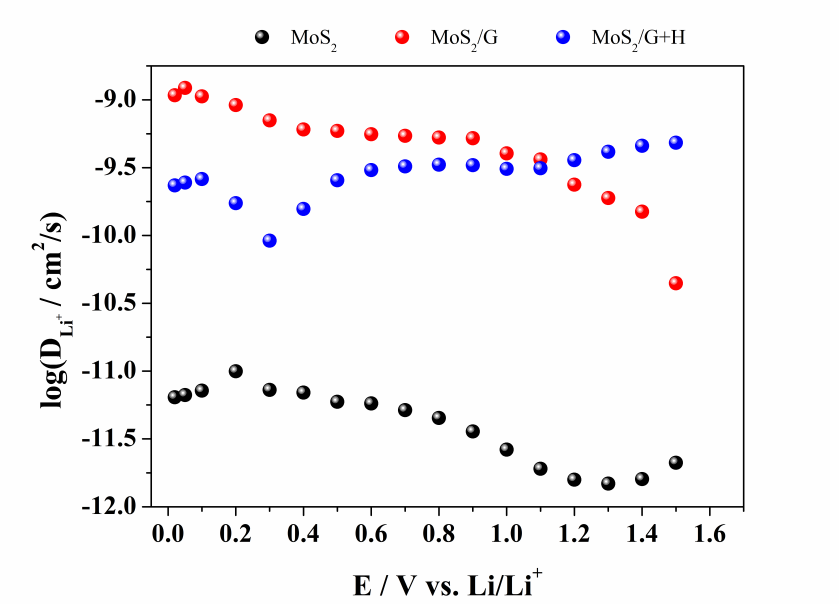


**Fig. S7.** Comparison of Li-ion diffusion coefficient for MoS_2_-based electrode materials.


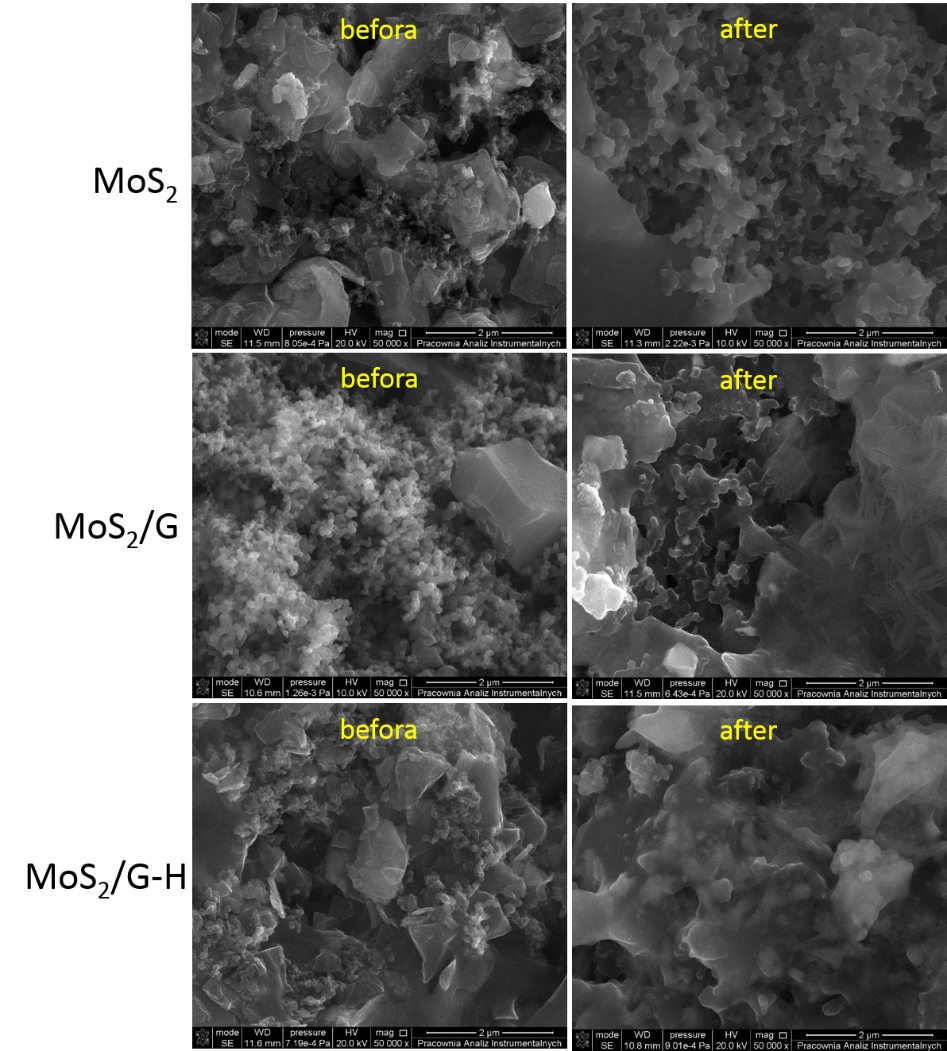


**Fig. S8.** SEM images of the obtained material layers on copper before and after electrochemical measurements in Li-ion batteries.


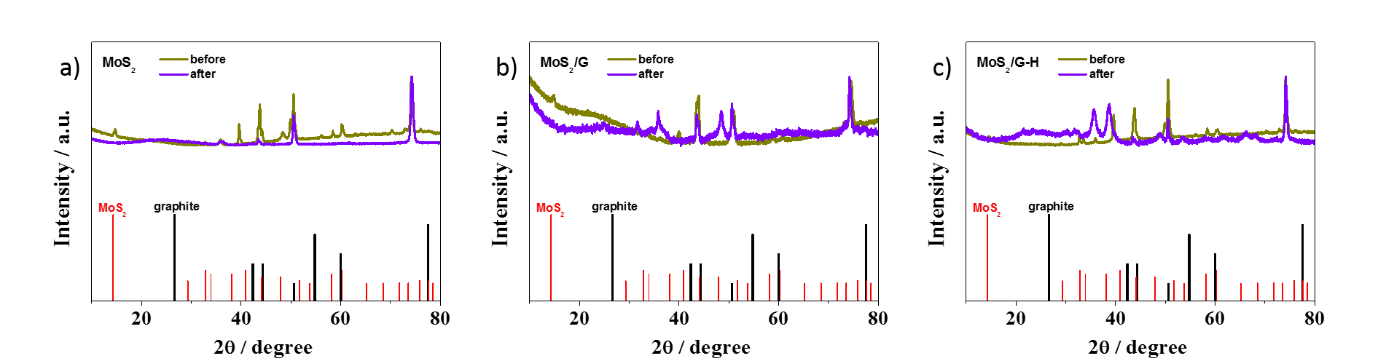


**Fig. S8.** XRD of the obtained material layers on copper before and after electrochemical measurements in Li-ion batteries.
